# Synergistic Structuring and Stabilization of Arid Agro-Soils via a Binary Metacid–Chitosan Interpolymer Complex

**DOI:** 10.3390/polym18182265

**Published:** 2026-09-17

**Authors:** Moldir Kerimkulova, Aitugan Sabitov, Kuanyshbek Musabekov, Gulmira Issenova, Orynkul Yessimova, Nurai Nurlan, Karagoz Seilkhan, Didar Kapantaikyzy

**Affiliations:** 1 Department of Analytical, Colloid Chemistry and Technology of Rare Elements, Al-Farabi Kazakh National University, Al-Farabi Ave., 71, Almaty 050040, Kazakhstan; moldir.kerimkulova@kaznu.edu.kz (M.K.); musabekov40@mail.ru (K.M.); orinkul.esimova@kaznu.edu.kz (O.Y.); nuraikamereika@gmail.com (N.N.); karagozseilkhan@gmail.com (K.S.); 2 Institute of Natural Sciences, Kazakh National Women’s Teacher Training University, Gogol Str., 114, K. 1, Almaty 050000, Kazakhstan; 3 Institute of Combustion Problems, Bogenbay Batyr 172, Almaty 050012, Kazakhstan; 4 Testing Center for Phytosanitary Laboratory Analysis, Kazakh Research Institute of Plant Protection and Quarantine, Kultobe Str., 1, Almaty 050070, Kazakhstan; isenova.gd@gmail.com; 5 Department of Science, International IT University, Manas 34/1, Almaty 050040, Kazakhstan; dbolatova@iitu.edu.kz

**Keywords:** interpolymer complex, soil structuring, polyhexamethylene guanidine hydrochloride, chitosan, anti-erosion resistance, rheological properties, zeta potential, wind deflation, synergistic effect

## Abstract

This study investigates the effectiveness of using an interpolymer complex based on a synthetic polycation—Metacid (polyhexamethylene guanidine hydrochloride) and a natural biopolymer—chitosan for anti-erosion soil structuring in the Maktaaral district of the Turkestan region. Using the conical plastometry method, the critical concentration of structure formation in the soil suspension was determined to be 67.5%; for subsequent studies, the solid phase content was taken to be 70%. Rheological studies have revealed a pronounced synergistic effect of the combined use of Metacid and chitosan. If the initial soil–water system is characterized by low elastic moduli (E1, E2 ~ 10^4^ Pa), then the introduction of the “Metacid–chitosan” interpolymer complex leads to a sharp strengthening of the structure: The values of E1 and E2 increase to 3.26·10^6^–7.49·10^6^ Pa and 1.04·10^6^–1.46·10^6^ Pa, respectively. The mechanical stability limit increases by more than twofold, reaching 2000–2500 Pa. Analysis of the ζ-potential and electrical conductivity showed that the Metacid ensures a high positive charge of the complex (+21.76 … 28.69 mV), and chitosan acts as a spatial matrix. Experiments in a wind tunnel have confirmed that the “Metacid–chitosan” formulation, at an optimal concentration of 0.4%, provides effective protection of soil aggregates against wind erosion. The results obtained open up new prospects for using ecologically balanced interpolymer complexes to stabilize arable lands.

## 1. Introduction

Erosion poses a serious threat to the national economy and the environment, as it has a profound destructive impact on the state of soil cover, which is the foundation of agricultural production and a crucial element of the biosphere. The development of erosion processes is determined by a complex range of factors, including natural and climatic conditions (climate, terrain, genetic type and granulometric composition of soils), as well as anthropogenic activity related to the intensive use of land resources in the agricultural sector [1,2]. Depending on the leading physical mechanism of soil profile destruction, wind erosion (deflation) and water erosion are traditionally distinguished.

Wind erosion represents a critical environmental and agricultural challenge in Kazakhstan, where over 24 million hectares of arable and pasture lands are affected by soil degradation [3,4]. The problem is particularly pronounced in the arid regions of Southern Kazakhstan, such as the Turkestan region, where intensive cotton and melon cultivation under high ambient temperatures and moisture deficits leads to severe structure deterioration of the topsoil layer. Preventing the loss of fine-grained fertile fractions under extreme climatic conditions necessitates the development of effective chemical approaches for anti-erosion stabilization.

The use of high-molecular-weight compounds is considered one of the most promising and technologically advanced methods for protecting soils from degradation [5,6]. The physicochemical effect of polymer stabilizers on the dispersed soil system is realized through three key colloidal mechanisms:

(a) Bridging flocculation and aggregation: long macromolecular chains are adsorbed on the surface of soil colloids, acting as a molecular adhesive and binding erosion-prone fine-dispersed fractions into strong, water-resistant, and wind-resistant macroaggregates.
(b) Optimization of infiltration: securing structural elements prevents hydrodynamic destruction of clumps and the formation of a dense surface “crust” of compaction, which blocks the penetration of moisture. This increases water permeability, promoting water absorption and reducing surface runoff.
(c) Formation of a protective coating: applying polymer solutions to the surface creates a thin, elastic three-dimensional polymer–soil matrix that shields the soil from the kinetic energy of raindrops and the tangential stress of wind flows.

Synthetic polyacrylamide (PAA) and its anionic derivatives are widely used in agricultural and geotechnical practices to stabilize soil aggregates and reduce sediment transport [7,8,9,10]. However, the stabilization efficacy of PAA strongly depends on soil texture, application method, and slope gradient, often requiring high application rates or combination with organic mulches to achieve sufficient control [11,12,13]. Furthermore, the continuous large-scale utilization of PAA is constrained by critical environmental concerns, including the potential release of toxic monomeric acrylamide—a known neurotoxin and carcinogen—and the exceptionally slow biodegradation rate of the polymer matrix in soil (<10% per year), leading to its progressive environmental accumulation.

Natural biopolymers are being actively studied as an environmentally friendly alternative to synthetic polymers, in particular chitosan—an aminopolysaccharide derived from renewable raw materials and subject to complete biodegradation [14,15]. Comparative tests in irrigation canals showed that although in laboratory conditions chitosan reduced sediment transport by more than 85% (which is comparable to PAA), in full-scale field experiments its effectiveness dropped to 51%, compared to 99% for PAA. This fact is due to the rapid enzymatic degradation of individual chitosan by soil microflora and fluctuations in its conformational state caused by changes in pH and ionic strength of soil solutions.

Chitosan contains active functional groups, including free amino groups and hydroxyls. In a weakly acidic environment, the amino groups undergo protonation to form a soluble cationic form, converting chitosan into a polyelectrolyte with a high density of positive charge [16].

Due to their cationic nature, chitosan macromolecules are capable of intense electrostatic binding with negatively charged surfaces of soil colloids (including clay minerals, quartz, and humus), as well as forming inter-particle macromolecular “bridges” and hydrogen bonds [17]. Studies show that adding chitosan solutions can significantly increase the shear modulus, ultimate compressive strength, and shear resistance of loosely cohesive sandy and silty soils due to effective pore filling and “gluing” of grains [18].

However, the use of individual chitosan for soil erosion control has a number of serious limitations. Chitosan is insoluble in water at neutral and alkaline pH values, requiring weak organic acids for dissolution. In highly carbonate-rich, alkaline soils (such as arid gray soils) or in the near-isoelectric region, the protonation of amino groups decreases, which leads to conformational changes, chain folding, a drop in the density of the positive charge, and a decrease in the mechanical-physical stability of aggregates. The addition of dry powder forms of chitosan often leads to the loosening of the soil matrix and a certain increase in porosity, while the maximum strengthening effect is achieved only when using gel/dissolved forms [17].

An individual biopolymer is susceptible to relatively rapid enzymatic degradation under the influence of soil microflora, and the interparticle bonds formed by it may undergo gradual leaching and softening in a moist environment. To overcome the shortcomings of individual polymers, a fundamentally new class of structure-forming agents—interpolyelectrolyte (interpolymer) complexes (IPCs)—has been proposed in the last decade [19,20]. IPCs are formed as a result of cooperative electrostatic, hydrogen, and hydrophobic interactions between oppositely charged macromolecules and are amphiphilic copolymers with extended hydrophilic and hydrophobic blocks. Possessing the ability to bind complementarily with the active sites of silicate minerals, IPCs effectively “cross-link” the dispersed phase [21]. Varying the molar ratio of components and the architecture of the IPC allows for precise tuning of the mechanical strength, water resistance, and biodegradation kinetics of protective coatings [22,23], while simultaneously ensuring moisture retention and prolonging the effect of nutrients.

Thus, to combat soil erosion in agricultural soils in the Republic of Kazakhstan, an urgent scientific and practical task is to develop and implement polycomplex systems based on biodegradable polymers from renewable sources, which demonstrate increased efficiency under harsh climatic conditions.

Previously, functional materials containing polyhexamethyleneguanidine and chitosan were studied primarily as antibacterial surface coatings for biomedical dressings or paper additives [24,25]. However, applying such binary polyelectrolyte systems to heterogeneous soil environments represents a fundamentally different colloidal domain. Unlike solid textile substrates, erodible arid soils require in situ complexation to anchor onto charged clay minerals, alter electro-surface boundary potentials, and cross-link dispersed mineral grains into an anti-deflationary framework. To address these challenges, this study investigates the physicochemical principles of anti-erosion stabilization of arid agro-soils (Maktaaral district, Turkestan region) using a binary interpolymer complex (IPC) composed of synthetic polycation Metacid and natural biopolymer chitosan. The main objective is to establish the synergistic structuring mechanism of the binary complex and its capacity to form a mechanical- and water-resistant soil–polymer framework. Specifically, this study aims to: (1) determine the critical concentration of structure formation and quantify the rheological behavior of modified soil suspensions; (2) elucidate electro-surface interactions (ζ-potential and electrical conductivity) at the soil–polyelectrolyte interface; and (3) evaluate the aerodynamic stability of soil aggregates under controlled wind-tunnel conditions. Combining a high-charge synthetic polycation with a spatial matrix of a natural polysaccharide provides a compelling laboratory proof-of-concept for stabilizing highly erodible arid arable soils. While this study establishes the fundamental colloidal and aerodynamic feasibility of the IPC binder under controlled conditions, evaluating its practical agricultural applicability requires addressing real-world field dynamics.

## 2. Materials and Methods

### 2.1. Characteristics of the Original Soil

The main object of the study was samples of arid soil collected from the territory of the LLP “Agricultural Experimental Station of Cotton and Melon Growing,” located in the village of Atakent, Maktaaral District, Turkestan Region, Republic of Kazakhstan. The samples were taken from the arable horizon (depth 0–20 cm) using the envelope method. Before conducting the experiments, the soil was dried to an air-dry state, cleared of plant residues, and sifted through a sieve with a 1 mm hole diameter to isolate the erosively hazardous fine-dispersed fraction.

### 2.2. Characteristics of Polymer Modifiers

Two water-soluble polymers with complementary molecular weights and macromolecular dimensions were used to structure the soil:

(a) Metacid (polyhexamethylene guanidine hydrochloride)—a synthetic, highly charged polycation with an average molecular weight of M_w_ ≈ 12–15 kDa (degree of polymerization N ≈ 67–84). Theoretical estimations indicate a fully extended contour length (*L_max_*) of 80–100 nm and a compact coil radius of gyration (R_g_) of 2–4 nm in aqueous media. This compact coil size and low molecular weight ensure high conformational mobility, rapid dissolution, and unhindered diffusion into submicron clay pores, enabling fast electrostatic anchoring onto negatively charged mineral surfaces. Working solutions of Metacid were prepared by dissolving an accurate weight of the polymer in distilled water.
(b) Chitosan—a natural aminopolysaccharide derived from crustacean shells (manufactured by LLC “Bioprog”, Moscow, Russia), with an average molecular weight of M_w_ ≈ 200 kDa and a degree of deacetylation of 82–85%. In contrast to Metacid, high-molecular-weight chitosan exhibits a substantially larger coil hydrodynamic dimension (*R_g_* ≈ 40–70 nm) and extended chain persistence length. This high molecular weight was specifically selected to facilitate long-range inter-particle bridging across macro-voids and to build a flexible spatial polymer matrix capable of enmeshing pre-flocculated soil aggregates into a coherent three-dimensional network [26,27].

Due to the insolubility of chitosan in a neutral aqueous medium, its working solutions were prepared by dissolving the sample in a 1% acetic acid solution under magnetic stirring for 24 h until complete dissolution and a stable pH (4.5–5.0) were achieved.

The binary interpolymer complex (IPC) “Metacid–chitosan” is formed via cooperative electrostatic interactions and hydrogen bonding between the cationic guanidinium groups of Metacid and the amino/hydroxyl centers of chitosan. The complex was prepared by controlled mixing of individual polymer working solutions at room temperature (22 ± 2 °C) under intensive magnetic stirring for 1 h to ensure complete polyelectrolyte complexation. The total dosage of binary compositions, calculated per dry mass of soil, ranged from 0.1% to 1.0%.

### 2.3. Methods of Experimental Research

FTIR spectra of the samples were recorded using a spectrometer Cary 630 FTIR, (Agilent, Penang, Malaysia) in the wavenumber range of 400–4000 cm^−1^ in KBr pellets.

The electrosurface characteristics of the systems were assessed by measuring the electrokinetic potential and specific electrical conductivity. The measurements were carried out using a Zetasizer Nano ZS instrument (Malvern Panalytical, Malvern, United Kingdom) at a temperature of 25 °C in triplicate (n = 3, with 10–15 sub-runs per measurement). For analysis, diluted aqueous suspensions of soil particles treated with polymers and their binary composition were prepared within the studied concentration ranges.

X-ray phase analysis was carried out using an automated diffractometer DRON-4 (JSC “Bourevestnik”, St. Petersburg, Russia) using the powder diffraction method (the radiation was recorded in the angular range θ from 10° to 70°). Cu anode, operating parameters: 40 kV, 40 mA, increment 0.02°, scan speed 1.0 s/s with double accumulation to improve the signal-to-noise ratio. Qualitative identification of crystalline phases and quantitative calculation of the mineral composition were carried out using specialized software EVA v 4.1 and PCPDFWIN v 2.4, with the involvement of the international PDF-2 diffraction database. The chemical composition of the soil was determined by X-ray fluorescence analysis (XRF). The studies were conducted using a high-precision wavelength-dispersive X-ray fluorescence spectrometer of the Axios series (PANalytical, Almelo, The Netherlands), which allows for the precise quantitative determination of the content of the main rock-forming and glass-forming oxides. The spectrum was recorded with a semiconductor detector made of ultrapure Si and cooled using the Peltier effect. The work was carried out in accordance with the procedure described in [28].

### 2.4. Determination of the Critical Concentration of Structure Formation and Plastic Strength

The critical concentration of structure formation (CCS) of soil suspensions was determined using a Rebinder conical plastometer KP-3 (Crypto LLC, Dzerzhinsk, Russia). The method is based on measuring the depth of cone penetration (h) into the soil paste under the influence of a given constant load (F). The plastic strength (Pm) was calculated using Rebinder’s formula:

Pm = K_α_ × F/h^2^

where K_α_ is the cone constant, which depends on the angle at its apex. Experiments were conducted by varying the moisture content (solid phase concentration) of soil pastes from 40% to 80% to construct structure formation curves and identify the inflection point [29].

### 2.5. Rheological Analysis of the Sample Structure

To evaluate the structural–mechanical and rheological properties of soil pastes, polymer solutions (or IPC mixtures) were added to air-dry soil (sieved through a 1 mm mesh). The system was gently mixed by hand using a stainless steel spatula for 3–5 min until a homogeneous paste with a fixed solid-phase content of 70% (selected based on the established critical concentration of solutes—67.5%) was obtained. Intensive mechanical shearing or grinding was intentionally avoided to prevent mechanical crushing of primary mineral particles and changes in the initial particle size distribution. The complex structural–mechanical and elastic–plastic–viscous properties of modified soil systems with a solid phase content of 70% were studied using a rotational viscometer (Reotest-2) in the mode of a given shear stress.

Based on the kinetic curves of strain development over time (ε = f(t)) under constant stress and the subsequent drop in strain after the load is removed, the following fundamental rheological constants were calculated using the Hooke–Newton–Saint-Venant model [30]:

1. Fast elastic modulus (E_1_);
2. Slow elastic modulus (E_2_);
3. Equilibrium elastic modulus (E);
4. Maximum plastic viscosity (η);
5. Conditional limit of dynamic flow (P_k1_);
6. Relaxation period (θ).

The interpolymer complex “Metacid–chitosan” was prepared by mixing working solutions at a 3:1 mass ratio. This ratio was selected based on solution phase behavior, application requirements, and literature precedents for soil-stabilizing polyelectrolyte complexes [17,19,20,25]. Preliminary mixing of individual working solutions showed that higher chitosan proportions (e.g., 1:1 or 1:2) led to excessive solution viscosity and premature macro-phase separation (coacervate precipitation) prior to application. Maintaining a 3:1 excess of Metacid ensured solution stability and low working viscosity suitable for uniform pneumatic spraying, while providing sufficient cationic charge density to anchor onto negatively charged mineral surfaces, consistent with optimal IPC formulations reported for soil aggregation [19,20].

The mixing was carried out at room temperature (22 ± 2 °C) under conditions of intensive stirring on a magnetic stirrer IKA myPlate (IKA-Werke GmbH & Co. KG, Staufen, Germany) for 1 h. The total concentration of the polymer mixture, calculated based on the absolutely dry mass of the soil, varied within the range from 0.1% to 1.0%. The required total moisture content of the soil pastes (70%) when applying the IPC solution was achieved by adding the calculated volume of distilled water.

### 2.6. Tests for Soil Resistance to Wind Deflation

**Figure 1:**
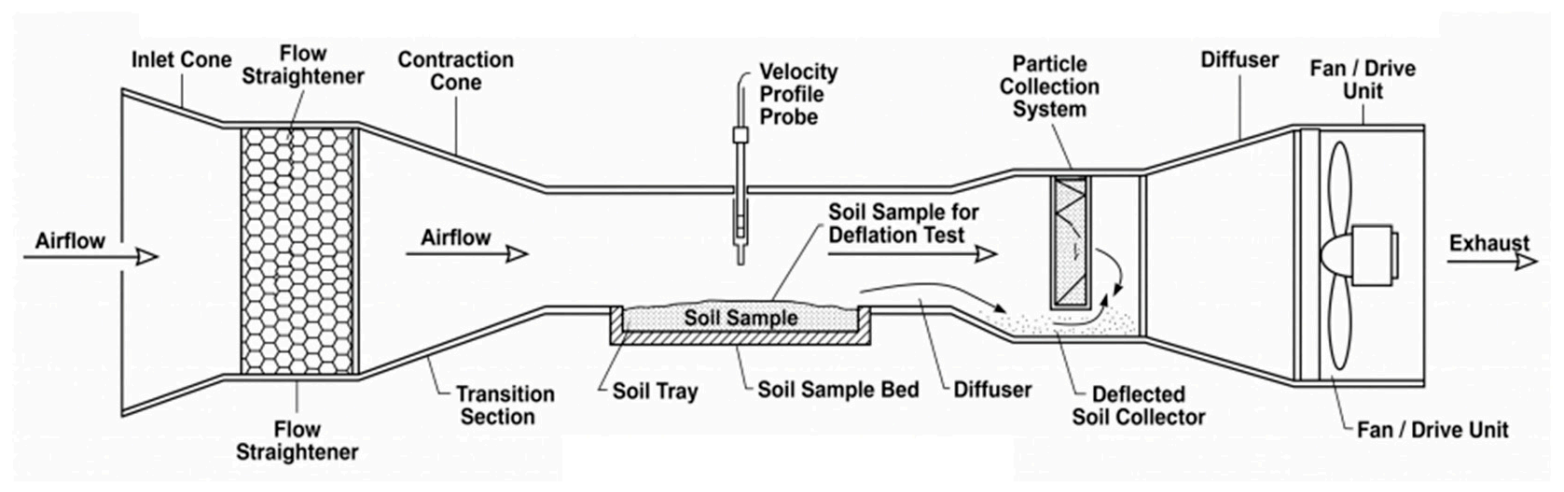
Diagram of a specialized laboratory wind tunnel (wind tunnel) for analyzing wind erosion of soil.

The diagram of the laboratory complex for conducting deflation tests is shown in Figure 1. The open-type wind tunnel includes a flow equalization section (a honeycomb straightener and a coupling nozzle), a working chamber with a tray for the soil sample, a system for separating and collecting suspended particles, and an adjustable ventilation unit.

Wind erosion tests were performed using air-dry soil samples (surface area S = 0.025 m^2^, layer thickness 20 mm, particle fraction <1 mm) prepared in specialized testing trays. To simulate realistic field application and preserve the natural bulk particle-size distribution of the soil, the polymer binders and IPC formulations were applied via uniform pneumatic surface spraying at a rate of 1.5 L/m^2^, rather than bulk mechanical mixing. This surface application method generates a continuous, elastic polymer–soil protective crust (1.5–2.5 mm thick) via capillary imbibition and surface adsorption, leaving the sub-surface grain structure undisturbed. Treated samples were air-dried at 22 °C to constant mass prior to aerodynamic testing.

The sample was placed in the working section of the pipe flush with its lower base to exclude edge-related turbulent effects. The air flow was supplied in steps with a step of 1–3 m/s within the range from 5 to 15 m/s (the duration of each step was 3 min). The flow velocity and the boundary layer profile above the sample surface were monitored using a Pitot tube and a digital pressure gauge. The intensity of wind deflation was calculated based on the mass of soil particles carried away and captured in the cyclone per unit of time, relative to the sample surface area:

q = Δm/(S·t)

where Δm is the loss of sample mass (g), S is the area of the exposed soil surface (m^2^), t is the duration of exposure to the wind flow (s) [31]. All aerodynamic wind-tunnel tests were performed in triplicate (n = 3) for each sample and wind velocity step.

The physical characteristics of the resulting protective surface crust were evaluated after full air-drying of the sprayed soil samples. The crust thickness was measured on cross-sectional fractures of the treated soil layer using a digital vernier caliper (with an accuracy of ±0.01 mm) across ten randomly selected points per tray to determine the mean thickness (1.5–2.5 mm).

## 3. Results and Discussion

### 3.1. The Mineralogical, Phase, and Structural-Group Composition of the Original Soil

To gain a detailed understanding of the colloidal-chemical mechanisms of interaction between structure-forming polymers and the dispersed matrix of the soil, the phase composition and surface functional groups of the initial sample of gray soil from the Maktaaral district of the Turkestan region (Atakent village) were comprehensively studied at the first stage.

**Figure 2:**
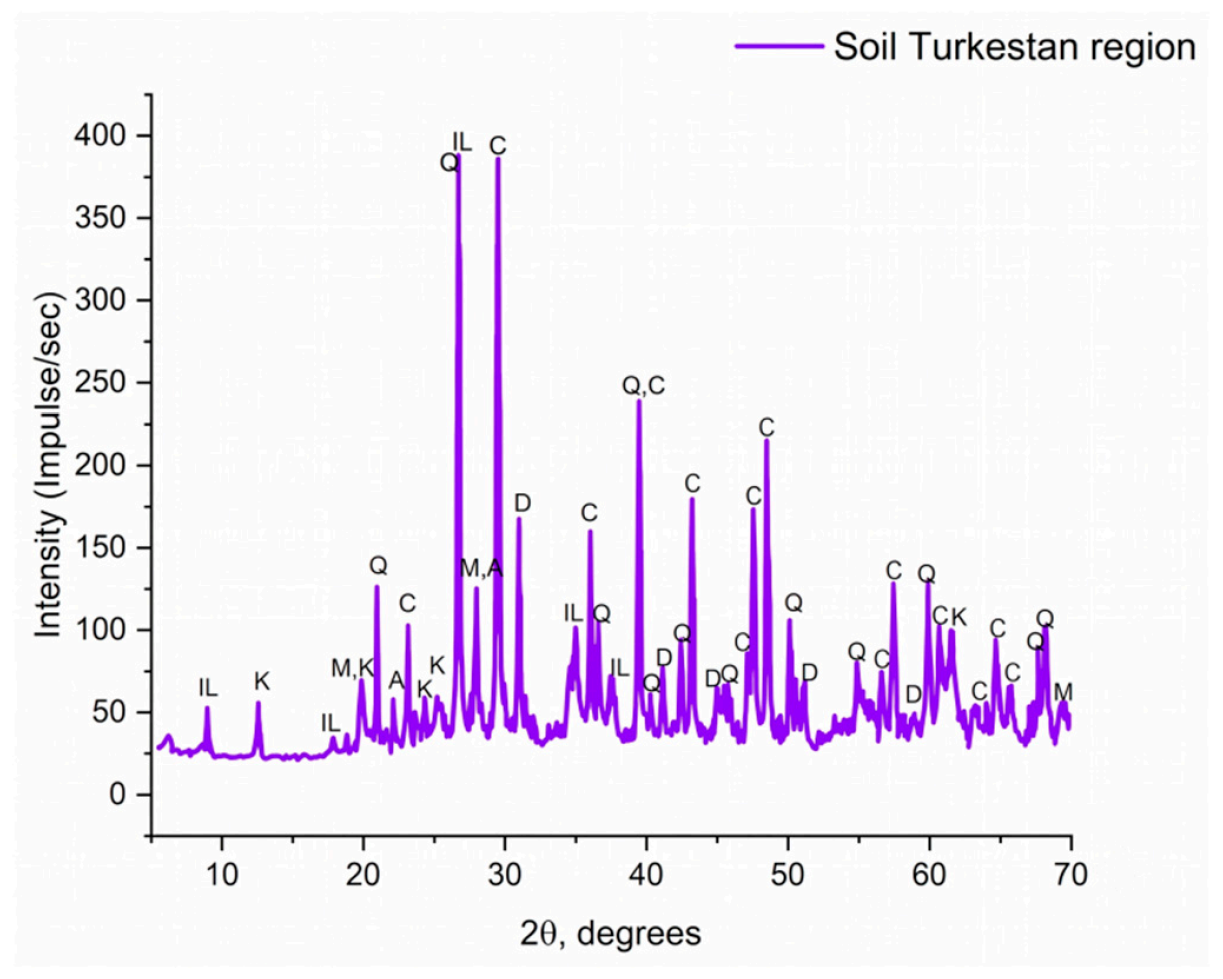
XRD spectrum of the soil sample: IL—illite, K—kaolinite, M—muscovite, Q—quartz, A—albite, C—calcite, D—dolomite.

The results of qualitative and quantitative X-ray phase analysis (XRD), presented in Figure 2 and Table 1, indicate the polymineral composition of the soil under study:

(a) Carbonate complex (Calcite and Dolomite): The total proportion of carbonate minerals in the sample under study is 31.68%. The dominant phase is calcite (CaCO_3_)—24.80%, and the content of dolomite (CaMg(CO_3_)_2_) is recorded at 6.88%. A high degree of carbonation is typical for gray soils in arid zones of Kazakhstan. The presence of calcite in the X-ray diffraction pattern is confirmed by a series of intense diffraction reflections (marked with the index C), among which the most pronounced peak is in the region 2θ ~ 29.4°. The presence of a significant amount of calcite and dolomite determines the buffer properties of the soil solution and the alkaline reaction of the medium, which is of critical importance for the subsequent conformational state of the introduced polyelectrolytes.
(b) Clay and mica minerals (Illite, Kaolinite, Muscovite): The highly dispersed aluminosilicate fraction, which determines the soil’s sorption and flocculation activity, is represented by illite (27.34%), kaolinite (10.65%), and muscovite (10.20%). The total content of clay and mica components reaches 48.19%. On the XRD spectrum, the presence of illite and kaolinite is clearly identified by basal reflections in the low-angle region (2θ ~ 8.8° for illite (IL) and 2θ ~ 12.4° for kaolinite (K)). The layered structure of these minerals is characterized by the presence of a constant negative excess charge on the basal planes (due to isomorphic substitutions in tetrahedra and octahedra), as well as by ragged cleavage planes on the periphery of the crystals, which contain amphoteric hydroxyl groups ≡Si-OH and ≡Al-OH. It is precisely these negatively charged planes that act as the main adsorption centers for cationic macromolecules [32,33].
(c) Framework silicates (Quartz and Albite): Quartz (SiO_2_) accounts for 13.68% of the total mass, while sodium feldspar—albite (NaAlSi_3_O_8_)—accounts for 0.45%. Quartz produces characteristic narrow and intense diffraction maxima at 2θ ~ 20.8°, 26.6°, and 36.5°. These minerals form a rigid, skeletal framework of the soil, but they have a relatively low specific surface area and surface charge density compared to the clay fraction.

To confirm the elemental composition of the sample, X-ray fluorescence analysis (XRF) was performed; the results are presented in Table 2.

The data from XRF analysis are strictly consistent with the results of phase analysis (Table 1). The high content of silicon dioxide (48.20%) and aluminum oxide (11.40%) is due to the predominance of an aluminosilicate framework (illite, kaolinite, muscovite, quartz). Calcium oxides (14.80%) and magnesium oxides (3.45%), together with calcination losses (13.32%), reflect a significant proportion of carbonate minerals (31.68% calcite and dolomite), which form the alkaline buffer of the soil environment. The presence of K_2_O (2.85%) confirms the presence of an illite fraction, which acts as the main adsorption center for the cationic chains of polymers [34].

**Figure 3:**
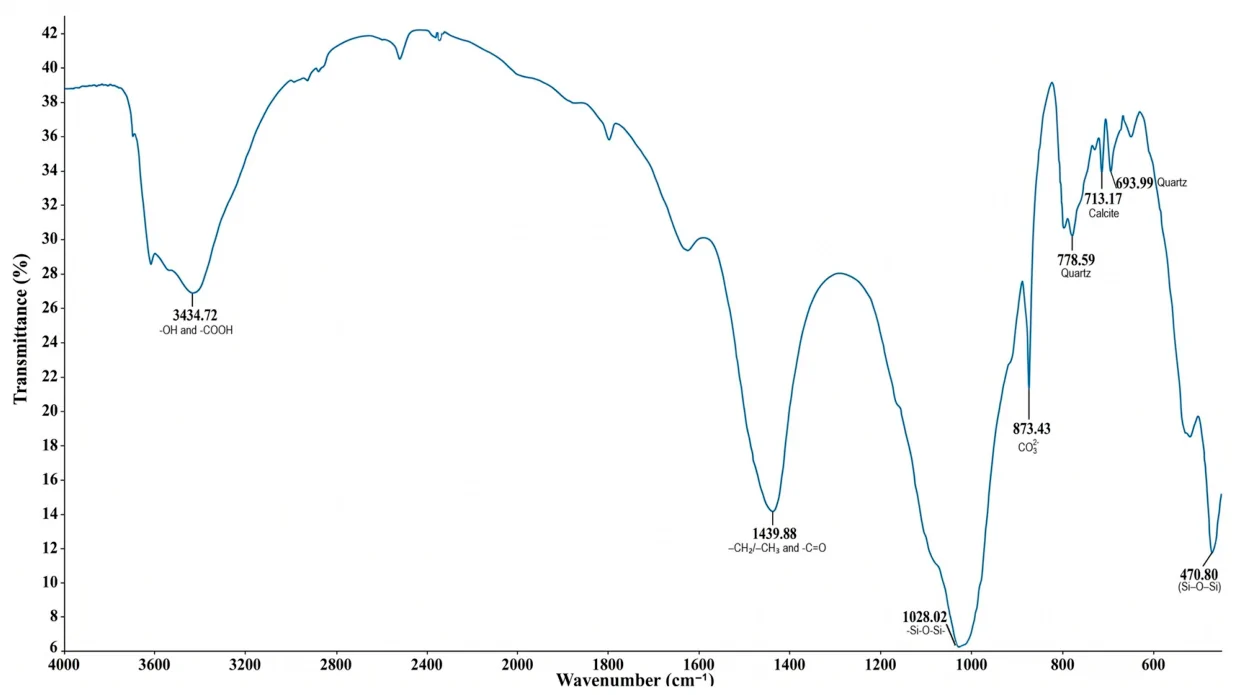
FTIR spectrum of the original soil sample from the Maktaaral district.

To identify the surface functional groups responsible for subsequent binding to polyelectrolytes, the FTIR spectrum of the initial sample of soil was analyzed in detail (Figure 3). The resulting spectrum reflects the complex heterogeneous structure of the soil matrix, consisting of a mineral base and organic matter (humus).

The presence of humic acids (humic and fulvic acids) in the soil of the arid zone of the Turkestan region makes a decisive contribution to several key areas of the spectrum. The FTIR spectrum of the soil matrix (Figure 3) reveals characteristic absorption bands of both the mineral base and humic substances. The broad band centered at 3434 cm^−1^ corresponds to the overlapping valence vibrations of alcoholic/phenolic –OH and carboxyl –COOH groups of humic and fulvic acids involved in hydrogen bonding. The intense band at 1439 cm^−1^ reflects a superposition of aliphatic –CH_2_/–CH_3_ deformation vibrations from organic matter and C–O carbonate vibrations of calcite and dolomite (>31% of the mineral phase). The dominant band at 1028 cm^−1^ is assigned to asymmetric Si–O–Si stretching in illite, kaolinite, and quartz, where its frequency shift confirms the presence of organo-mineral complexes anchored via divalent cation (Ca^2+^, Mg^2+^) bridges. Characteristic carbonate groups of calcite (873 cm^−1^, 779 cm^−1^) and quartz doublet (778 and 694 cm^−1^) bands further corroborate the XRD data [35,36].

### 3.2. Determination of the Critical Concentration of Structure Formation

**Figure 4:**
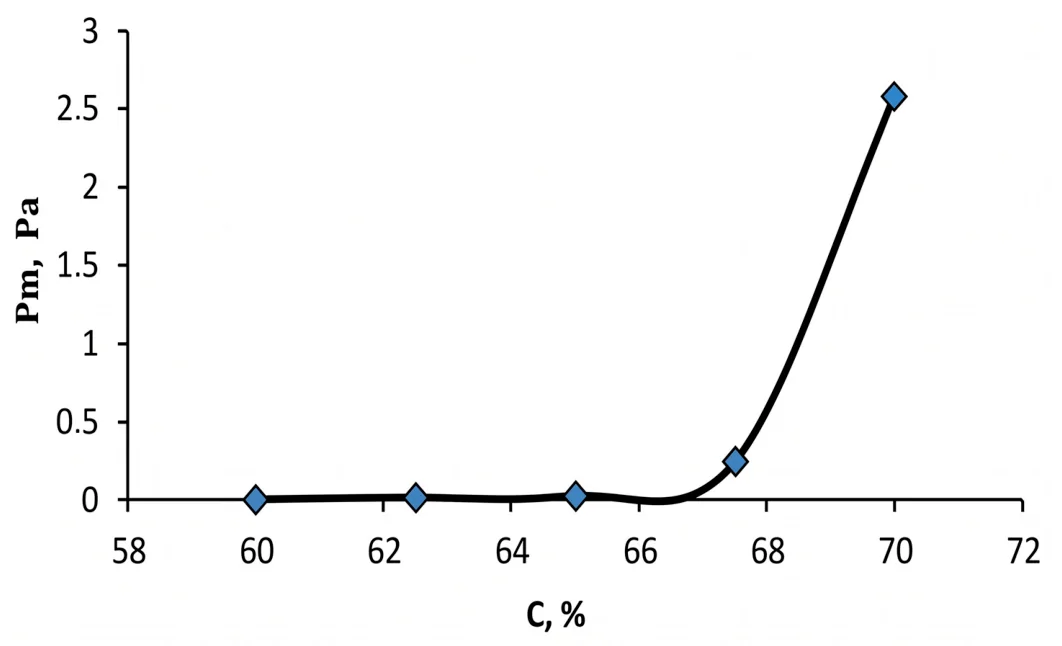
Critical concentration of structure formation in clean soil.

To study the structural and mechanical constants and indicators, it was necessary to determine the critical concentration of soil structure formation. The critical concentration of structure formation (CCS) for the unamended soil suspension, determined via Rebinder conical plastometry, was established at 67.5% solid phase (Figure 4). For all subsequent experiments, a fixed solid-phase content of 70% was selected to model an optimal elastic–plastic system capable of maintaining shear stresses.

**Figure 5:**
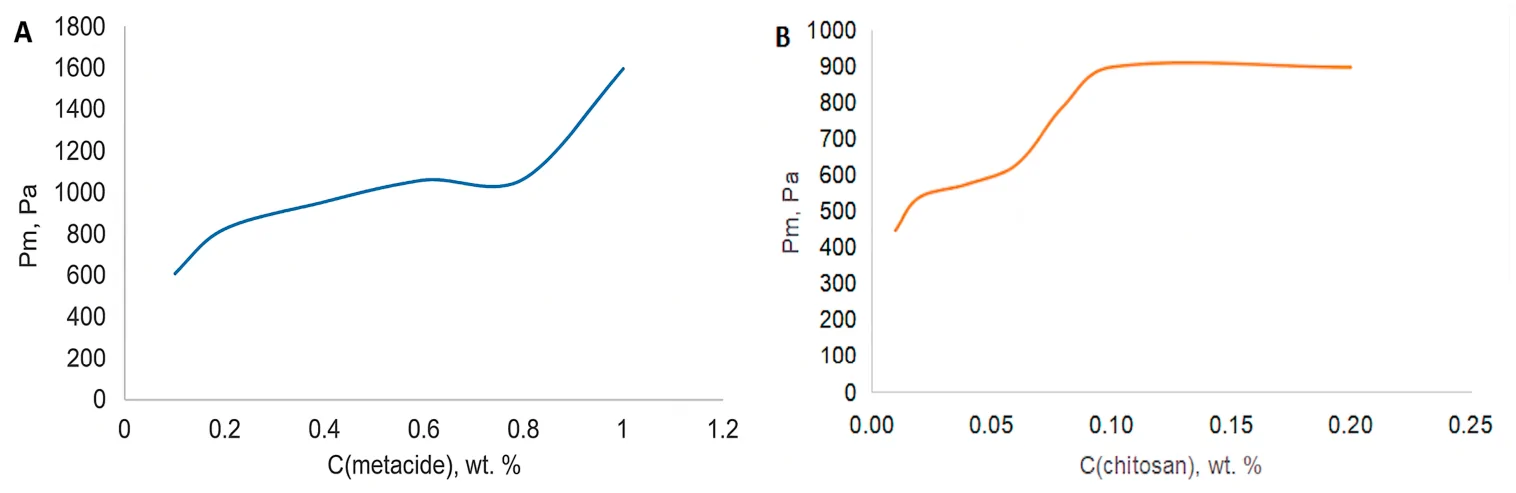
Effect of polymer concentration on the plastic strength (Pm) of the soil framework (70% solid phase): (**A**) Metacid; (**B**) chitosan.

Modification of the 70% soil suspension with individual Metacid (0.1–1.0%) leads to a progressive increase in plastic strength (P_m_) from 612.9 to 1059.8 Pa up to a polymer dosage of 0.6% (Figure 5A). This initial strengthening is driven by bridging flocculation, where highly charged polycation chains adsorb onto negatively charged basal planes of illite and kaolinite, as well as humic carboxylate sites. The local strength plateau observed within the 0.6–0.8% range corresponds to charge compensation and partial steric repulsion between similarly charged floccules. Beyond 0.8%, the steep exponential rise in P_m_ (reaching 1593.6 Pa at 1.0%) marks a structural transition from bridging flocculation to a bulk polymer–mineral gel composite formed under constrained moisture conditions.

In contrast, high-molecular-weight chitosan (0.01–0.2%) displays a classic S-shaped adsorption isotherm (Figure 5B). Initial neutralization of surface charges elevates P_m_ from 446.2 to 537.4 Pa at 0.02%, followed by a sharp increase to 896.3 Pa at 0.1% due to extended macromolecular bridging across soil aggregates. Reaching a strict horizontal plateau beyond 0.1% indicates complete adsorption saturation of the active organo-mineral sites, where excess biopolymer provides steric protection without further structural compaction.

### 3.3. Structural and Mechanical Properties of Soil Systems Based on Rotational Viscometry Data

Table 3 presents the fundamental structural and mechanical constants and rheological characteristics of the original unmodified “soil–water” system with a solid phase content of 70%, selected based on the established critical concentration (67.5%).

The obtained data quantitatively characterize the initial elastic–plastic–viscous state of the soil dispersion. The fast and slow elastic moduli (E_1_ and E_2_) are 45,131.5 Pa and 10,745.59 Pa, respectively, and the calculated equilibrium elastic modulus E is 8676.71 Pa. The value of the conditional limit of dynamic flow (P_k1_) is 1000 Pa, which reflects the weakness of the coagulation framework of the untreated soil. The highest plastic viscosity η is 2.26·106 Pa·s, and the relaxation period (θ), calculated using the Maxwell model, is 260.46 s. The established parameters indicate that plastic deformations prevail over elastic ones and determine the high vulnerability of the arable layer to destructive external loads, which requires the use of effective polymeric structure-forming agents. Since the aim of the work was to observe the effect of structure-forming agents on soil in order to enhance the effect of these agents on eroded soils, a study was conducted to examine the effect of the solutions on the soil, first individually and then in combination.

### 3.4. Synergistic Structural–Mechanical Behavior of Soil Hydrosuspensions Modified with Metacid, Chitosan, and Their Binary Interpolymer Complex

Modifying soil suspensions with polymer binders significantly transforms their elastic–plastic–viscous behavior. Table 4, Table 5 and Table 6 summarize the structural–mechanical constants calculated using the Hooke–Newton–Saint-Venant model for systems with a solid phase content of 70%.

As shown in Table 4, increasing Metacid concentration from 0.1% to 0.6% monotonically increases the instantaneous (E_1_) and equilibrium (E_2_, E) elastic moduli, alongside the plastic viscosity (η) which peaks at 6.02·10^7^ Pa·s (at 0.6%). This structural strengthening originates from polycation adsorption onto aluminosilicate planes and humic carboxylates, forming a continuous coagulation network.

It is interesting to note the dynamics of the yield strength (P_k1_). While P_k1_ for the initial unamended soil is 1000 Pa, its value temporarily decreases to 400–900 Pa at low to moderate Metacid concentrations (0.2–0.6%). This effect is attributed to alterations in electro-surface characteristics: the primary adsorption of the highly charged polycation leads to the neutralization of the surface charge of soil particles and the compression of the double electrical layer, causing a temporary plasticization of the paste and facilitating relative shear displacement of the floccules. However, upon reaching concentrations of 0.8–1.0% and forming a bulk polymer-mineral gel, P_k1_ increases back to 1100 Pa.

Accordingly, the relaxation period (θ) increases from 284 s to 5330 s, reflecting the strengthening of the internal structure and the deceleration of its destruction under shear stresses. At the same time, the elasticity parameter (Λ) monotonically decreases from 0.05 to 0.02, indicating a transition of the system from a mobile plastic state to an elastically rigid structured composite.

With a further increase in Metacid concentration beyond the optimum (within the range of 0.8% to 1.0%), a slight decrease in viscosity and elastic moduli is observed (viscosity decreases to 3.91·10^7^ Pa·s). This effect is explained by the achievement of adsorption saturation on the soil particle surface with polymer macromolecules and their complete steric blockade, leading to a slight loosening of the spatial network due to polyelectrolyte excess and the predominance of solvation processes [37,38].

Thus, the optimal concentration of Metacid for effective soil structuring in the Turkestan region is 0.6%, which ensures the achievement of maximum strength and viscosity characteristics of the dispersed system.

For the soil–chitosan system (Table 5), adding high-molecular-weight biopolymer (0.01–0.1%) induces a sharp rise in viscosity up to 6.50 × 10^7^ Pa·s (at the optimal concentration of 0.1%). Such a sharp increase in rheological parameters is probably due to the high density of reactive amino and hydroxyl groups in chitosan macromolecules, which provide effective bridging of dispersed soil particles and the formation of a strong coagulation–crystallization network [26].

Accordingly, the relaxation period (θ), reflecting the strength and durability of the internal structure, increases from 283 s to 6820 s. The elasticity parameter (Λ) decreases from 0.05 to 0.01, which indicates stabilization of the system and the predominance of elastic–plastic properties over purely plastic deformations. With a further increase in polymer concentration to 0.2%, a decrease in viscosity and a reduction in elastic moduli are observed. This effect is explained by the achievement of adsorption saturation of the surface of soil aggregates with polysaccharide macromolecules and the oversaturation of the system, which leads to steric hindrance, partial coiling of polymer coils, and a certain loosening of the spatial framework.

A comparison of the effectiveness of polymers with different molecular weights shows that high-molecular-weight chitosan (200 kDa) reaches the maximum level of structuring at significantly lower concentrations (0.1%) than relatively low-molecular-weight Metacid (15 kDa, with an optimum at 0.6%). This is due to the fact that long macromolecular chains of chitosan have a greater capacity to form extended adsorptive-solvate bridges between soil particles, whereas shorter chains of Metacid require a higher concentration to form a spatial framework of similar density.

To assess the nature of the formation of coagulation-thixotropic structures and the strength properties of soils in the Turkestan region when they are treated with a binary polymer system, fundamental rheological constants and characteristics were calculated using the Hooke–Newton–Saint-Venant model (Table 6).

**Figure 6:**
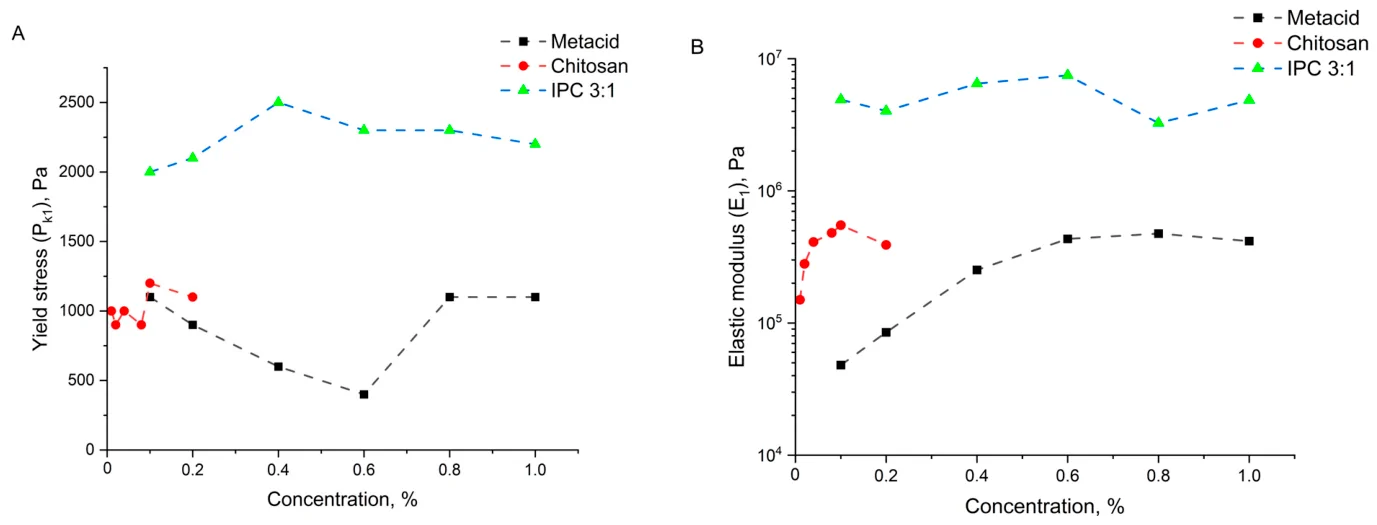
Comparative analysis of structural–mechanical behavior of soil hydrosuspensions (70% solid phase) modified with Metacid, chitosan, and binary IPC (3:1 ratio): (**A**) conditional yield stress (Pk1) as a function of polymer concentration; (**B**) instantaneous elastic modulus (E1) on a logarithmic scale.

A profound non-linear synergistic effect is quantitatively demonstrated by employing the binary IPC “Metacid–chitosan” at a 3:1 mass ratio (Figure 6). Mathematically, mechanical synergy is established as the IPC parameters (X_IPC_) vastly exceed the additive sum of the individual components. At the optimal dosage of 0.40%, the IPC elevates the conditional yield stress (*P_k_*_1_) to 2500 Pa (Figure 6A), representing a >108% mechanical increase over the individual maxima of Metacid (1100 Pa) and chitosan (1200 Pa).

Even more dramatically, the instantaneous elastic modulus (E_1_) of the IPC system reaches (3.26–7.49)·10^6^ Pa, representing a tenfold increase (>1100%) over single-polymer formulations (4.75–5.50·10^5^ Pa) (Figure 6B). This non-additive increase quantitatively confirms the formation of an ionically cross-linked macromolecular polycomplex network within the soil matrix. Concurrently, the marked decrease in relaxation period (θ = 12.86–60.6 s) compared to individual polymers (θ = 284–6820 s) confirms a fundamental shift from slow entanglement-driven relaxation to rapid stress distribution within a rigid, ionically cross-linked three-dimensional polymer–mineral framework.

### 3.5. The Dependence of Soil Deflation Intensity on Wind Flow Speed and the Composition of Modifiers

**Figure 7:**
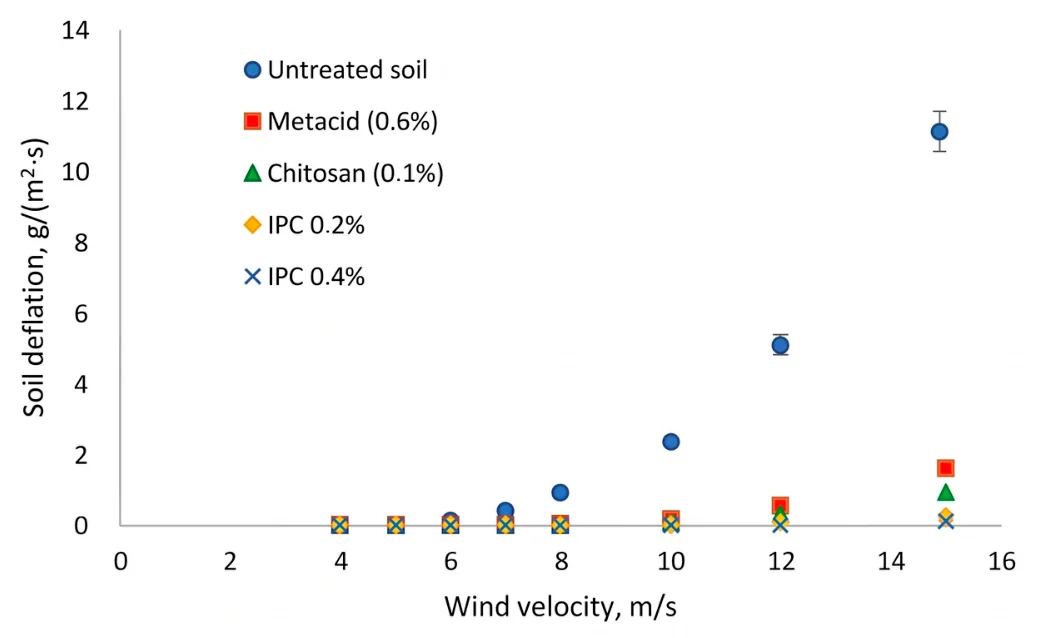
The relationship between the intensity of soil deflation and the wind flow velocity when treated with individual polymers and their combination.

An analysis of the kinetics of wind erosion (Figure 7) showed that for unprocessed soil, intensive mass loss begins at a threshold wind flow velocity of 5 m/s. At a speed of 15 m/s, the deflation intensity of the control sample reaches 11.145 g/(m^2^s), which indicates complete destruction of the bonds between unprocessed soil aggregates.

Modifying the soil with individual polymer binders significantly shifts the threshold for the onset of deflation and reduces mass loss. At the maximum test speed (15 m/s), Metacid (0.6%) reduces the carry-over rate to 1.650 g/(m^2^s), while high-molecular-weight chitosan (0.1%) reduces it to 0.940 g/(m^2^s). The data from aerodynamic tests are in strict agreement with the results of plastometry and rotational viscometry (Table 4, Table 5 and Table 6), where the maximum plastic strength and shear strength (P_k1_ = 2500 Pa) are also achieved within the 0.4% interval.

However, the greatest anti-erosion effect is achieved when using the binary IPC “Metacid–chitosan”. Figure 7 clearly shows the profound synergistic effect of their combined action. Introducing an IPC with a concentration of 0.2% reduces mass loss at 15 m/s to 0.280 g/(m^2^ s).

Increasing the concentration of IPC to 0.4% ensures almost complete aerodynamic stability of the surface layer: the deflation rate drops to 0.120 g/(m^2^s), which corresponds to a reduction in wind erosion by more than 98.9% compared to the control sample. Cross-sectional morphological examination revealed that pneumatic spraying of the 0.4% IPC solution forms a continuous, cohesive polymer–soil surface crust with a mean thickness of 1.8 ± 0.3 mm. The capillary imbibition of the IPC solution cross-links fine soil grains into a durable three-dimensional matrix, providing high structural resistance against wind flow without altering the underlying bulk particle size distribution. At this optimal IPC dosage, complete filling of the active adsorption sites on the surface of aluminosilicate minerals (illite, kaolinite) and humic substances is achieved.

The experimental curve of IPC (0.4%) in Figure 7 practically merges with the x-axis. Further increase in the concentration of the complex does not result in a noticeable change in erosion, as the deflation values reach a physical plateau within the measurement error range of ≤0.02 g/(m^2^s).

### 3.6. Studying the Influence of Modifier Composition on the Surface Potential of Soil Particles

The electrosurface characteristics of soil suspensions (electrokinetic ζ-potential and specific electrical conductivity) serve as a fundamental indicator of the colloidal-chemical mechanism of interaction between polymeric reagents and the negatively charged surface of aluminosilicates and humus. The measurement results are presented in Table 7.

When Metacid is introduced within the concentration range of 0.1–0.4%, an instantaneous and profound recharge of the soil surface potential is observed, shifting from negative values to consistently positive ones (the ζ-potential increases to +41.8 … +44.36 mV). This is explained by the high density of positively charged guanidine groups in Metacid, which are intensely electrostatically adsorbed onto the basal planes of clay minerals (illite, kaolinite) and carboxylate ions of humus.

With a further increase in the concentration of Metacid to 1.0%, the ζ-potential gradually decreases to +36.97 mV, which is accompanied by a linear increase in the electrical conductivity of the system from 0.7181 to 5.014 mS/cm. This effect is associated with the saturation of adsorption centers in soil fractions, the accumulation of excess free macroions and counter-ions (Cl^−^) in the volume of the soil solution, and the compression of the double electric layer due to an increase in the ionic strength of the medium [39].

Modification with a weak polyelectrolyte (chitosan). The behavior of chitosan is fundamentally different and demonstrates the classic curve of electrostatic neutralization. Within the range of minimum dosages (0.01–0.04%), ζ-potential remains in the negative region (from −13.58 to −13.92 mV).

An intensive process of surface charge neutralization unfolds within the range of 0.08–0.1%, where the ζ-potential shifts sharply from -9.68 mV to the point of the isoelectric state (IES at C = 0.1%, ζ = −0.61 mV). It was at this concentration that an extremum of plastic viscosity and elastic moduli was recorded in rheological studies, which proves that reaching the IES promotes maximum coagulation and flocculation of soil particles due to the removal of electrostatic repulsion forces. Further addition of chitosan (0.2%) leads to a slight recharging of the surface to +0.63 mV. The electrical conductivity increases slightly (to 0.523 mS/cm), reflecting the weakly ionogenic nature of the biopolymer [40].

**Figure 8:**
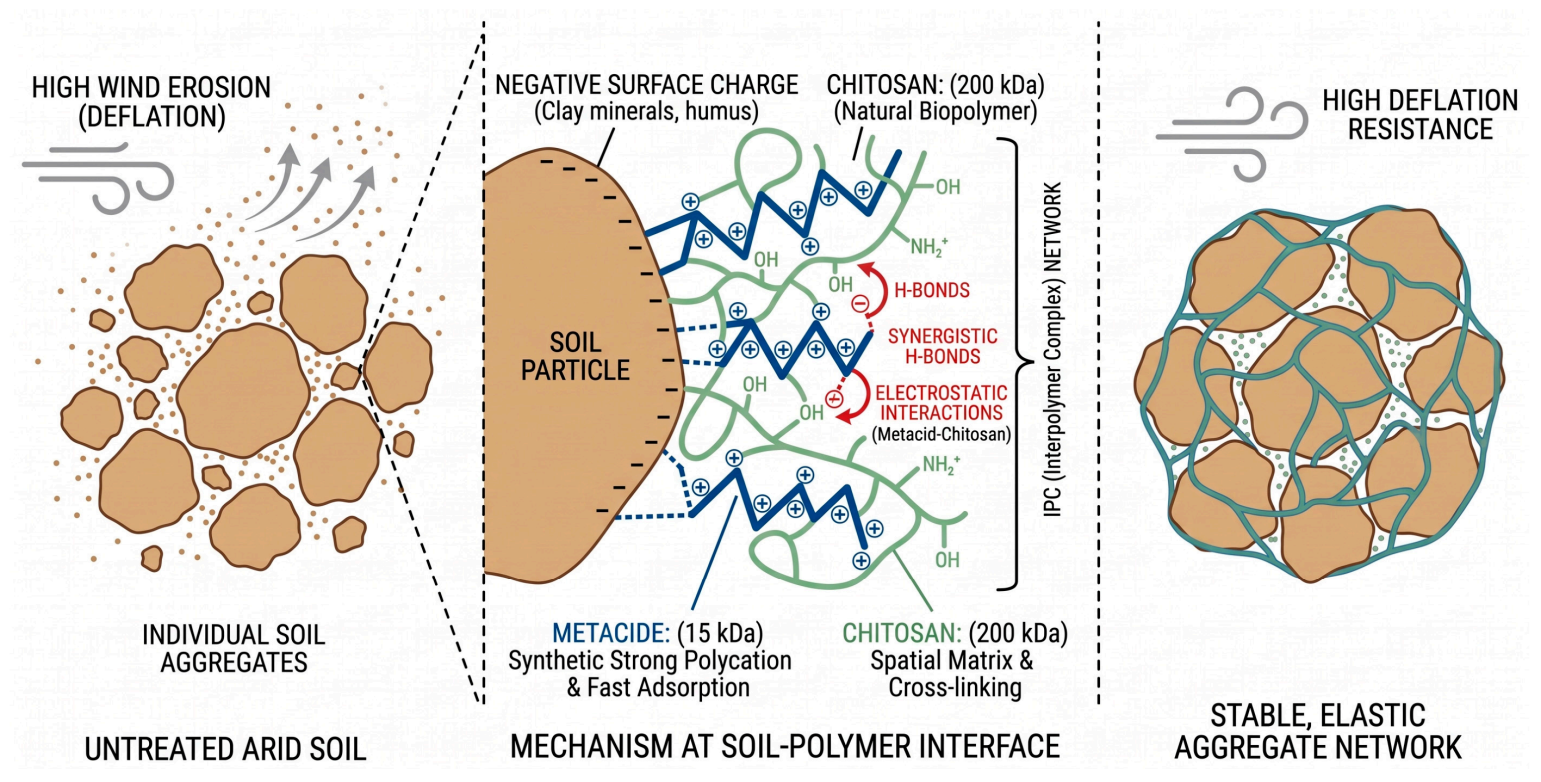
Scheme of the mechanism of soil structuring by IPC “Metacid–chitosan”: electrostatic fixation on the surface of minerals and formation of a spatial network to increase anti-erosion resistance.

Modification with a binary interpolymer complex (Metacid/Chitosan 3:1). The formation of the binary “Metacid–chitosan” system yields a stable positive electrokinetic potential across the entire studied concentration range (+15.72 … +28.69 mV), operating through a cooperative polyelectrolyte complexation mechanism. Strong physical–chemical evidence for in situ interpolymer complex (IPC) formation and coacervation is provided by the electro-surface and rheological measurements. Specifically, as the IPC dosage increases from 0.1% to 1.0%, the specific electrical conductivity drops monotonically from 1.852 down to 0.9545 mS/cm (Table 7). This substantial decrease in ionic mobility directly reflects the cooperative binding of oppositely interacting macromolecular segments and the entrapment/screening of counterions (Cl^−^) within the dense coacervate matrix. In this IPC network, Metacid acts as a highly charged polycation driving rapid electrostatic anchoring onto negatively charged aluminosilicate basal planes, while high-molecular-weight chitosan provides spatial chain entanglements that cross-link soil aggregates into an elastically reinforced 3D framework (Figure 8).

Of particular interest is the dynamics of the specific electrical conductivity of the IPC: while at the initial concentration (0.1%), the conductivity is 1.852 mS/cm, as the dosage of the complex increases to 0.4–1.0%, a consistent decrease is observed to 0.9545 mS/cm. This directly confirms the binding of counterions and the formation of a compact, interpolymer coacervate on the surface of soil aggregates, where low electrical conductivity at 0.4% (1.485 mS/cm) and a stable ζ-potential (+23.08 mV) guarantee the formation of a durable, insoluble organomineral framework.

Comparing the electrosurface parameters with the laws of the classical DLVO theory allows for a deeper understanding of the structuring mechanism. For the original soil, the ζ-potential value is -18.4 mV, which results in moderate electrostatic repulsion between particles and weak aggregation in the untreated suspension.

When an individual Metacid is introduced, an increase in the ζ-potential to +44.36 mV shifts the system into the region of high colloidal stability of similarly charged flocculates, which explains the slight plasticization of the soil paste at moderate dosages. In the case of chitosan, by contrast, passing through the isoelectric point (at C = 0.1%, ζ = −0.61 mV) completely neutralizes the electrostatic repulsion barrier, initiating auto-flocculation and achieving the maximum plastic strength.

On the curve of the binary IPC, at a concentration of 0.2%, a local minimum of the ζ-potential (+15.72 mV) is recorded, correlating with a sharp drop in specific conductivity to 1.659 mS/cm. This effect corresponds to the point of stoichiometric electrostatic cross-linking of polyelectrolytes with the surface of minerals, where the maximum binding of chloride counter-ions occurs in the volume of the developing polycomplex coacervate. Further stabilization of the ζ-potential within the range of +21.76 … +28.69 mV at C = 0.4% indicates the formation of an optimal hydrophobic-hydration balance: the complex retains a moderate positive charge for strong adhesion to aluminosilicates, but does not oversaturate the soil solution with free ions (electrical conductivity decreases monotonically to 0.9545 mS/cm at 1.0%), which ensures the high anti-deflationary and heat-moisture resistance of the formed crust.

## 4. Conclusions

This study demonstrates that the binary Metacid–chitosan IPC at an optimal 3:1 mass ratio provides highly effective stabilization of arid carbonate-rich gray soil against wind erosion. The introduction of 0.4% IPC induces substantial mechanical strengthening of the spatial soil matrix, increasing the conditional yield strength (P_k1_) up to 2500 Pa and the highest plastic viscosity (η) up to 5.44 ·107 Pa·s. This structural synergy significantly outperforms the binding capability of individual polymer components.

Electrosurface measurements (ζ-potential and electrical conductivity) confirm a dual stabilization mechanism. The strong polycation Metacid provides rapid electrostatic anchoring onto negatively charged clay minerals and humic carboxylates, while high-molecular-weight chitosan forms a flexible three-dimensional matrix that cross-links soil aggregates into a durable organo-mineral framework.

Aerodynamic wind-tunnel testing verified that surface application of 0.4% IPC forms a continuous protective crust, reducing soil loss at a wind velocity of 15 m/s from 11.145 down to 0.120 g/(m^2^·s). Increasing the complex dosage beyond 0.4% yields no further improvement, indicating that the system reaches a surface saturation plateau. Furthermore, partial replacement of costly biopolymer chitosan with synthetic Metacid substantially lowers raw material costs while enhancing structural performance, making low-dosage (0.4%) IPC treatment economically scalable for agricultural arid lands.

It is important to emphasize that these laboratory findings serve as a fundamental proof-of-concept under controlled conditions. Translating this technology to full-scale agricultural practice requires bridging the gap between bench-top testing and field applicability. Future research must focus on field-scale trials to evaluate the long-term durability of the polymer–soil crust under natural precipitation, freeze–thaw cycles, solar UV degradation, and mechanical disturbance from tillage equipment, alongside assessing IPC biodegradation kinetics and rhizosphere ecotoxicity.

## Figures and Tables

**Table 1:** Phase composition of soil.

Sample	Illite	Kaolinite	Muscovite	Quartz	Dolomite	Calcite	Albite
Soil	27.34	10.65	10.20	13.68	6.88	24.80	6.45

**Table 2:** Elemental (oxide) composition of the original soil sample according to XRF analysis.

Component	Mass Fraction, %	Physicochemical Role in the System
SiO_2_	48.2	Quartz skeleton, tetrahedral layers of illite and kaolinite.
CaO	14.8	Calcite (CaCO_3_), dolomite; a source of Ca^2+^ cations.
Al_2_O_3_	11.4	Octahedral layers of clay minerals (illite, kaolinite).
Fe_2_O_3_	4.1	Iron oxides/hydroxides, isomorphic substitutions in micas.
MgO	3.45	Dolomite (CaMg(CO_3_)_2_), octahedra of illite.
K_2_O	2.85	Interlayer-fixed cations of illite and muscovite.
Na_2_O	1.2	Feldspars (albite NaAlSi_3_O_8_).
TiO_2_	0.68	Accessory titanium-containing minerals.
LOI	13.32	Losses during calcination: decarbonation (CO_2_), removal of the constitutional water of hydromicas, and combustion of humus.

**Table 3:** Structural–mechanical constants and soil characteristics.

Structural and Mechanical Constants and Characteristics	Soil/Water System
E_1_, Pa	45,131.50
E_2_, Pa	10,745.59
E, Pa	8676.7
P_k1_, Pa	1000
η, Pa·s	2.26·10^6^
θ, s	260.5
Λ	0.49
1/E_1_	2.22·10^−5^
1/E_2_	9.31·10^−5^
1/E	1.15·10^−4^
1/η, Pa^−1^·s^−1^	4.42·10^−7^

**Table 4:** Structural and mechanical constants and characteristics of the soil–Metacid system in the Turkestan region.

Structural and Mechanical Constants and Characteristics	The Soil–Metacid System					
	Metacid Concentration					
	0.1%	0.2%	0.4%	0.6%	0.8%	1.0%
E_1_, Pa	48,007.0	85,023.1	252,102.7	432,974.2	475,069.0	416,498.8
E_2_, Pa	10,811.0	10,922.5	11,504.1	11,662.1	10,034.8	8094.9
E, Pa	8771	9357.6	11,025.4	11,356.2	9827.2	7940.5
P_k1_, Pa	1100.0	900	600	400	1100	1100
η, Pa·s	2.5·106	4.5·10^6^	3.5·10^7^	6.02·10^7^	4.51·10^7^	3.91·10^7^
θ, s	283.4	478	3182	5330	4582	4934.0
Λ	0.05	0.04	0.03	0.02	0.02	0.01
1/E_1_	2.08·10^−5^	1.18·10^−5^	4.01·10^−6^	2.33·10^−6^	2.11·10^−6^	2.40·10^−6^
1/E_2_	9.26·10^−5^	9.17·10^−5^	8.73·10^−5^	8.62·10^−5^	1.05·10^−4^	1.24·10^−4^
1/E	1.14·10^−4^	1.04·10^−4^	9.13·10^−5^	8.85·10^−5^	1.02·10^−4^	1.26·10^−4^
1/η, Pa^−1^·s^−1^	4.00·10^−7^	2.22·10^−7^	2.86·10^−8^	1.66·10^−8^	2.22·10^−8^	2.55·10^−8^

**Table 5:** Structural and mechanical constants and characteristics of the soil–chitosan system in the Turkestan region.

Structural and Mechanical Constants and Characteristics	Soil–Chitosan System					
	Chitosan Concentration					
	0.01%	0.02%	0.04%	0.08%	0.1%	0.2%
E_1_, Pa	1.50·10^5^	2.80·10^5^	4.10·10^5^	4.80·10^5^	5.50·10^5^	3.90·10^5^
E_2_, Pa	9.20·10^3^	9.01·10^3^	9.20·10^3^	9.02·10^3^	9.70·10^3^	9.90·10^3^
E, Pa	8.67·10^3^	8.73·10^3^	8.99·10^3^	8.83·10^3^	9.53·10^3^	9.65·10^3^
P_k1_, Pa	1000	900	1000	900	1200	1100
η, Pa·s	2.45·10^6^	4.20·10^6^	1.50·10^7^	3.20·10^7^	6.50·10^7^	2.80·10^7^
θ, s	283	481	1668	3624	6820	2901
Λ	0.05	0.03	0.02	0.02	0.01	0.01
1/E_1_	6.67·10^−6^	3.57·10^−6^	2.44·10^−6^	2.08·10^−6^	1.82·10^−6^	2.56·10^−6^
1/E_2_	1.09·10^−4^	1.11·10^−4^	1.09·10^−4^	1.11·10^−4^	1.03·10^−4^	1.01·10^−4^
1/E	1.15·10^−4^	1.15·10^−4^	1.11·10^−4^	1.13·10^−4^	1.05·10^−4^	1.04·10^−4^
1/η, Pa^−1^·s^−1^	4.08·10^−7^	2.38·10^−7^	6.67·10^−8^	3.13·10^−8^	1.54·10^−8^	3.57·10^−8^

**Table 6:** Structural and mechanical constants and characteristics of the “Metacid–chitosan” system in the Turkestan region.

Structural and Mechanical Constants and Characteristics	The Concentration of the IPC “Metacid–Chitosan” (Ratio 3:1)					
	0.10%	0.20%	0.40%	0.60%	0.80%	1.00%
E_1_, Pa	4.90·10^6^	4.019·10^6^	6.48·10^6^	7.486·10^6^	3.258·10^6^	4.86·10^6^
E_2_, Pa	1.46·10^6^	1.22·10^6^	1.04·10^6^	1.30·10^6^	1.46·10^6^	1.29·10^6^
E, Pa	1.12·10^6^	9.33·10^5^	8.98·10^5^	1.11·10^6^	1.01·10^6^	1.02·10^6^
P_k1_, Pa	2000	2100	2500	2300	2300	2200
η, Pa·s	1.50·10^7^	1.20·10^7^	5.44·10^7^	5.61·10^7^	4.19·10^7^	3.38·10^7^
θ, s	13.39	12.86	60.6	50.6	41.6	33.0
Λ	0.23	0.23	0.14	0.15	0.31	0.21
1/E_1_	2.04·10^−7^	2.49·10^−7^	1.54·10^−7^	1.34·10^−7^	3.07·10^−7^	2.05·10^−7^
1/E_2_	6.84·10^−7^	8.22·10^−7^	9.60·10^−7^	7.68·10^−7^	6.85·10^−7^	7.70·10^−7^
1/E	8.90·10^−7^	1.07·10^−6^	1.11·10^−6^	9.01·10^−7^	9.93·10^−7^	9.75·10^−7^
1/η, Pa^−1^·s^−1^	6.67·10^−8^	8.33·10^−8^	1.84·10^−8^	1.78·10^−8^	2.39·10^−8^	2.96·10^−8^

**Table 7:** Results of the ζ-potential and electrical conductivity of soils modified with polymers and their complexes.

Metacid C,%	ζ-Potential (mV)	Electrical Conductivity (mS/cm)
0.1	41.8	0.7181
0.2	44.36	1.314
0.4	42.7	2.237
0.6	41.97	3.075
0.8	38.78	4.046
1.0	36.97	5.014
Chitosan C,%	ζ-potential (mV)	Electrical conductivity (mS/cm)
0.01	−13.58	0.2541
0.02	−14.69	0.2437
0.04	−13.92	0.2478
0.08	−9.684	0.2876
0.1	−0.6084	0.3721
0.2	0.6302	0.523
Metacid/Chitosan C,%	ζ-potential (mV)	Electrical conductivity (mS/cm)
0.1	25.98	1.852
0.2	15.72	1.659
0.4	23.08	1.485
0.6	21.76	1.286
0.8	28.22	1.114
1.0	28.69	0.9545

## Data Availability

The original contributions presented in this study are included in the article. Further inquiries can be directed to the corresponding author.

## Abbreviations

The following abbreviations are used in this manuscript:

IPC	Interpolymer Complex
PAA	Polyacrylamide
PGMH (Metacid)	Polyhexamethylene Guanidine Hydrochloride
IES	Isoelectric State
DLVO	Derjaguin–Landau–Verwey–Overbeek (theory)
XRD	X-Ray Diffraction
XRF	X-Ray Fluorescence
FTIR	Fourier-Transform Infrared (Spectroscopy)
CCS	Critical Concentration of Structure Formation
LOI	Loss on Ignition
PDF	Powder Diffraction File
UV	Ultraviolet
